# From pixels to pathology: how artificial intelligence mammographic risk scores capture tumor biology through imaging

**DOI:** 10.1007/s00330-026-12536-1

**Published:** 2026-04-21

**Authors:** Zi Zhang, Jafer Elabeid, Thowaiba Ali, Jennifer Pantleo, Nelda Gonzalez, Chirag Parghi

**Affiliations:** 1 Lewis Katz School of Medicine at Temple University, Philadelphia, PA USA; 2 Faculty of Medicine, University of Khartoum, Khartoum, Sudan; 3 Solis Mammography, Addison, TX USA

**Keywords:** Artificial intelligence, Breast neoplasms, Mammography, Risk assessment

## Abstract

**Objectives:**

To characterize clinical-pathologic tumor features associated with artificial intelligence (AI)–generated risk scores from prior-year screening mammograms.

**Materials and methods:**

This retrospective study included women who underwent breast biopsy following a screening mammogram in 2021 across four U.S. states. AI risk scores were obtained from prior-year screening mammograms using an FDA-approved AI model. Receiver operating characteristic (ROC) analysis was used to evaluate the discriminative ability of AI risk scores. Among patients with breast cancer, linear regression was used to assess associations between prior-year risk scores and cancer characteristics.

**Results:**

Among 1509 patients included (mean age 58.56 ± 12.28 years), 508 (33.7%) had biopsy-confirmed breast cancer. The area under the ROC curve (AUC) for prior-year AI risk score predicting biopsy-confirmed cancer was 0.62 (95% CI: 0.59–0.65). In univariate analysis of biopsy-positive patients, invasive lobular carcinoma (ILC) had significantly higher prior-year AI risk scores than ductal carcinoma in situ (*p* = 0.009), while Grade 3 tumors had significantly lower scores than Grade 1 (*p* = 0.016). After adjusting for tumor grade, the ILC association was no longer significant (*p* = 0.136), suggesting that tumor grade may mediate this relationship; Grade 3 tumors showed a marginal association with lower risk scores (*p* = 0.068).

**Conclusion:**

AI-generated risk scores from prior-year screening mammograms demonstrated modest discrimination between biopsy-confirmed malignant and non-malignant cases and may capture imaging features associated with low-grade tumors. Our findings suggest AI risk scores may reflect subtle imaging patterns of low-grade malignancy 1 year before clinical detection, thereby enhancing our understanding of AI model behavior and informing future research on clinical utility.

**Key Points:**

***Question*** *What clinical-pathologic tumor characteristics are reflected in artificial intelligence (AI)-generated risk scores from prior-year screening mammograms among patients with biopsy-confirmed outcomes?*

***Findings*** *Prior-year AI risk scores demonstrated modest discrimination between biopsy-confirmed malignant and non-malignant cases and may capture imaging features associated with low-grade tumors.*

***Clinical relevance*** *AI-generated risk scores from prior-year screening mammograms may capture subtle imaging features of evolving low-grade malignancy 1 year before clinical detection, offering insights into AI model behavior, enhancing explainability for clinicians, and informing future research on clinical utility.*

**Graphical Abstract:**

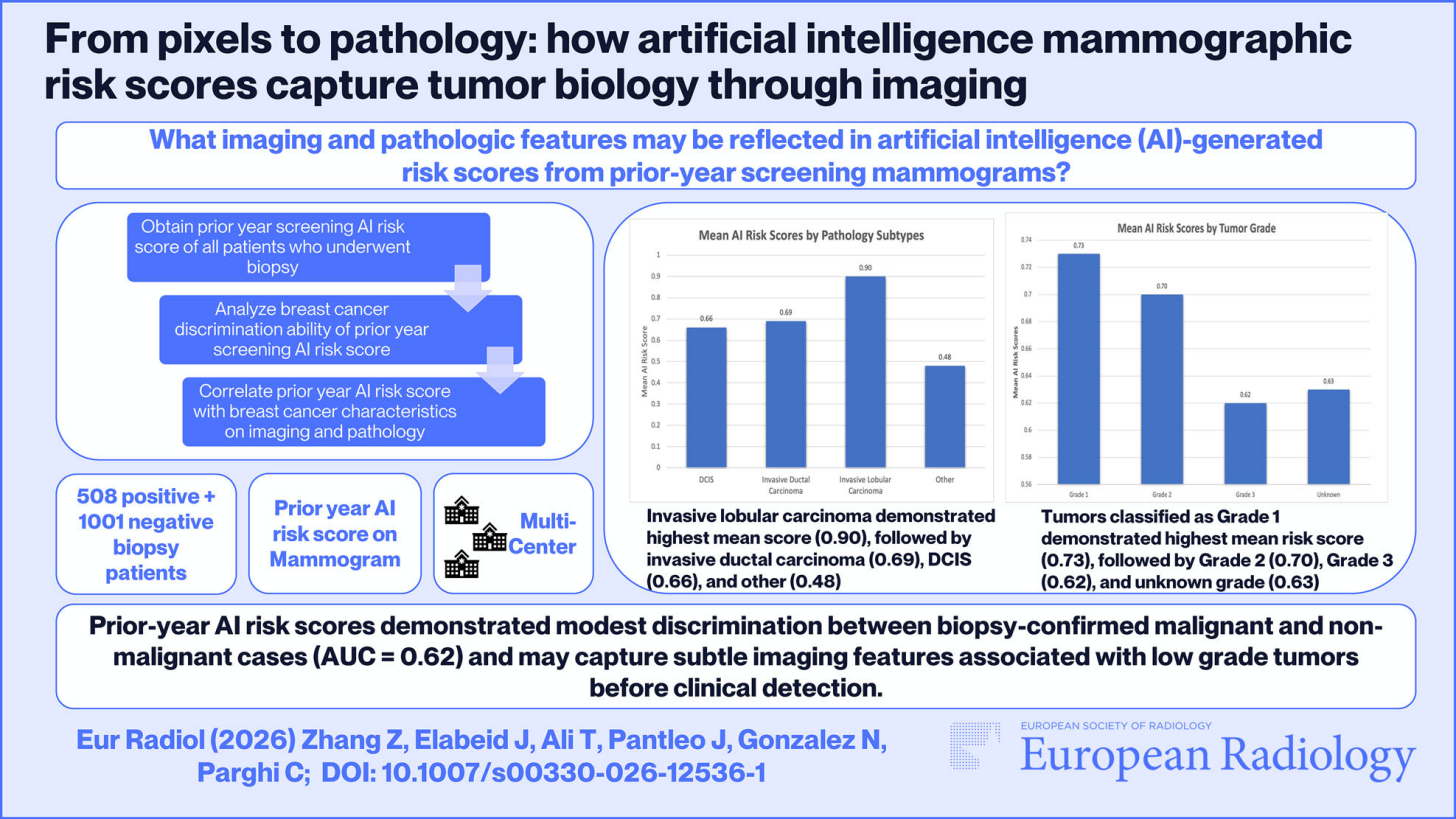

## Introduction

Breast cancer remains one of the leading causes of cancer-related morbidity and mortality among women worldwide [1]. Early detection through screening mammography significantly improves outcomes [2]; however, traditional risk assessment models, which often rely on demographic and clinical factors, have limited predictive accuracy and fail to capture the complex imaging biomarkers present in mammograms. Advances in artificial intelligence (AI) have introduced new opportunities to enhance breast cancer risk prediction by analyzing subtle imaging features that may precede clinical diagnosis [3, 4].

Recent AI models have demonstrated promising performance in estimating breast cancer risk from screening mammograms, often outperforming traditional clinical risk models [5]. While these studies have primarily focused on risk assessment at the time of imaging, limited research has explored how AI-predicted risk scores from prior screenings relate to biopsy-confirmed outcomes [6–8]. Moreover, the association between AI-generated risk assessments and clinical-pathologic features, such as tumor grade and pathologic subtypes, remains poorly understood [9–11]. Importantly, the “black box” nature of AI systems, wherein the underlying rationale for a risk prediction is not easily interpretable, limits clinical trust and transparency, making it essential to understand how these risk scores correlate with established biological and clinical features.

In this multicenter study, we evaluated how AI-generated risk scores from prior-year screening mammograms associated with biopsy outcomes and breast cancer characteristics. We also analyzed associations with pathologic subtypes, tumor grade, and receptor status to identify clinical and pathologic features linked to AI-estimated risk. By exploring potential biological correlates embedded in imaging data, our goal was to provide insights into the behavior of AI-based risk models and improve explainability and trust in these tools, especially in the context of “black-box” risk prediction AI models.

## Methods

### Study design and patient selection

This retrospective cohort study was conducted on patients who underwent screening mammograms across a multicenter network of breast imaging centers in the U.S. The study was approved by the Institutional Review Board, with the informed consent requirement waived due to the retrospective nature of the study.

Patients who underwent biopsy at our centers from January 1 to December 31, 2021, were identified through the PENRAD (Version 7.2) radiology and pathology reports. Demographic information included age, race/ethnicity, breast density and pathology reports (including pathologic subtype and receptor status) were also collected.

### Inclusion and exclusion criteria

We included all patients who underwent biopsy after receiving a BI-RADS 0 on screening mammography and a BI-RADS 4 or 5 on subsequent diagnostic imaging. Patients were eligible if they had a biopsy at one of our centers and had completed two consecutive years of screening mammography, specifically, a screening mammogram in the year prior to breast cancer diagnosis and another screening mammogram leading to the diagnosis in 2021.

Patients without screening mammography in the preceding year, patients with diagnostic rather than screening mammograms, and those without confirmatory biopsy with pathology were excluded.

### AI risk score and case score

AI-predicted risk scores were derived using the commercially available ProFound AI® Risk software (iCAD, Inc.), a U.S. Food and Drug Administration (FDA)-cleared deep learning–based algorithm designed for breast cancer risk assessment from screening mammograms. The ProFound AI system analyzes standard digital mammograms to quantify an individual’s short-term risk of being diagnosed with breast cancer, based on age, breast density and subtle mammographic features [12]. The risk score is a continuous numeric estimate of the patient’s risk of breast cancer relative to the entire female population, expressed as a percentage. Thus, the risk score can theoretically range from 0 to 100, although in practice the distribution is highly skewed toward lower values. These numeric percentage-based risk scores are stratified into Low, General, Moderate, and High-risk categories, with thresholds such as < 0.12 for Low and > 0.60 for High risk by the vendor, although categories may be locally calibrated [13]. The model has been previously validated for short-term risk prediction and demonstrated improved discrimination over traditional risk models in multiple large-scale cohorts [12, 14]. For our study, we extracted the AI risk scores from the prior-year screening mammogram of each patient. Additionally, AI risk scores from screening mammograms 2 years prior were extracted for patients diagnosed with breast cancer in 2021. All risk scores were generated as part of standard clinical workflow and exported for retrospective analysis.

In addition, AI case scores were obtained using ProFound Detection version 3.0 (iCAD Inc.), a companion FDA-approved algorithm developed for breast cancer detection. The algorithm evaluates all standard and supplemental mammographic views [15]. It assigns a case score from 0 to 100, representing the algorithm’s confidence that malignancy is present; higher scores reflect greater suspicion. For patients diagnosed with breast cancer in 2021, screening case scores were electronically retrieved from the year of diagnosis, as well as 1 and 2 years prior to diagnosis.

### Classification of breast density

Breast density was assessed using the Breast Imaging-Reporting and Data System (BI-RADS) criteria [16] as documented in the radiology report from the most recent screening mammogram. For analysis, breast density was categorized into two groups: non-dense (including breasts classified as almost entirely fatty or having scattered areas of fibroglandular density) and dense (including heterogeneously dense and extremely dense breasts).

### Classification of pathologic subtype

Pathology classifications were determined based on PENRAD queries of biopsy results. Breast cancer pathologies were classified as ductal carcinoma in situ (DCIS), invasive ductal carcinoma (IDC), invasive lobular carcinoma (ILC), and other invasive carcinoma (including mucinous, papillary, and medullary carcinomas). For patients with multiple breast cancer diagnoses, cases were manually reviewed to ensure that invasive malignancies were prioritized over in situ findings in the analysis. No cases included both IDC and ILC on biopsy. Additionally, non-breast malignancies incidentally identified on biopsy were excluded from the study.

### Classification of receptor status

Receptor status was determined based on immunohistochemistry (IHC) testing. Estrogen receptor (ER) and progesterone receptor (PR) positivity were defined as ≥ 1% nuclear staining, and human epidermal growth factor receptor 2 (HER2) positivity was defined as 3+ staining intensity on IHC or amplification confirmed by fluorescence in situ hybridization (FISH), following the American Society of Clinical Oncology/College of American Pathologists (ASCO/CAP) guidelines [17]. Invasive breast cancers were classified into four clinically relevant groups based on receptor status [18]: (1) HR positive/HER2 negative (Luminal A): ER and/or PR positive, HER2 negative; (2) HR positive/HER2 positive (Luminal B HER2+): ER and/or PR positive, HER2 positive; (3) HR negative/HER2 positive (HER2-enriched) HER2-enriched: ER and PR negative, HER2 positive; and (4) Triple-Negative Breast Cancer (TNBC): ER, PR, and HER2 all negative.

DCIS cases were reported separately based on hormone receptor [19] (ER and/or PR) status as either: 1. DCIS HR positive (ER and/or PR positive); or 2. DCIS HR negative (ER and PR negative). HER2 testing was not routinely performed for DCIS cases in accordance with standard clinical practice and was therefore not included in DCIS subgroup classification. Cases with incomplete or unavailable receptor information were classified as “unknown.”

### Statistical analysis

Descriptive statistics were used to summarize the data: continuous variables were reported as mean ± standard deviation, and categorical variables as frequencies and percentages. The Shapiro–Wilk test was used to assess the normality of continuous variables. Since the AI risk scores and case scores were not normally distributed (*p* < 0.001), nonparametric methods were employed where appropriate.

Statistical comparisons between groups were performed using appropriate tests based on variable type and distribution. For continuous variables, when the data followed a normal distribution, we used the independent samples *t*-test to compare means between groups. For non-normally distributed continuous variables, the Mann–Whitney U test was employed to compare medians. For categorical variables, we used the Chi-square (χ²) test of independence to evaluate differences in distributions between groups.

Because the distribution of prior-year risk scores was right-skewed and included zero values, we applied a natural logarithm (ln) transformation after adding a small constant (1 × 10⁻⁶) to all scores for all regression models.

We evaluated the discriminative performance of the AI-derived risk score from prior-year screening mammograms for breast cancer diagnosis using receiver operating characteristic (ROC) analysis. The area under the ROC curve (AUC) and its 95% confidence interval (CI) were calculated to quantify discrimination. To estimate the 95% CI of the AUC, we used the DeLong method, a nonparametric approach commonly employed for comparing correlated ROC curves and generating robust CI estimates.

Among patients with biopsy-confirmed breast cancer, we performed linear regression to assess associations between clinical and pathologic characteristics and AI-generated risk scores from prior-year screening mammograms. Univariable linear regressions were first conducted for age, race, breast density, cancer center location, pathology subtype, receptor status, and tumor grade. Variables with *p* < 0.05 in univariable analyses were then included in the multivariable linear regression model to adjust for confounding effects. All patients with biopsy-confirmed breast cancer were included in the multivariable linear regression. To minimize data loss, patients with missing pathology details, such as receptor type or grade, were categorized as ‘unknown’ and retained in the model. Regression results were reported as β coefficients with 95% confidence intervals and *p*-values.

#### Secondary analysis

To further evaluate clinical and pathologic characteristics associated with high AI risk scores on prior-year screening mammograms, we applied a binary threshold of > 0.60 based on vendor-defined cut points to define the high-risk group. Patients were categorized into a high-risk group (AI risk score > 0.60) and a lower-risk group (AI risk score ≤ 0.60).

A two-sided *p* < 0.05 was considered statistically significant. All statistical analyses were performed using STATA version 17.0.

## Results

### Associations between prior-year AI risk score and breast cancer diagnosis

Our study included a total of 1509 patients who underwent biopsy in 2021 and had prior-year screening mammograms across breast imaging centers located in Texas, North Carolina, Ohio, and Arizona. Among them, 508 (33.7%) had biopsy-confirmed breast cancer, while 1001 (66.3%) had benign findings. As shown in Table 1, the cancer group was significantly older, with a mean age of 66.75 ± 11.06 years, compared to 56.38 ± 11.81 years in the benign group (*p* < 0.001). Prior-year AI risk scores were significantly higher in the cancer group (mean 0.68 ± 0.54, median 0.57) than in the benign group (mean 0.48 ± 0.48, median 0.30) (*p* < 0.001). Among patients with a prior-year AI risk score > 0.60, nearly half (47.0%) were diagnosed with breast cancer, significantly higher than those (30.1%) in the lower risk group (*p* < 0.001).

**Table 1:** Patient demographics and prior-year AI risk scores by biopsy result

		Biopsy		
	Total	Positive for cancer	Negative for cancer	*p*-value
Number, *n* (%)	1509	508 (33.7)	1001 (66.3)	
Age, mean ± SD	58.56 ± 12.28	66.75 ± 11.06	56.38 ± 11.81	< 0.001
Prior-year case score				
Mean ± SD	48.20 ± 30.95	52.94 ± 31.30	45.79 ± 30.50	< 0.001
Median	44	52	43	< 0.001
Prior-year risk score				
Mean ± SD	0.55 ± 0.52	0.68 ± 0.54	0.48 ± 0.48	< 0.001
Median	0.36	0.57	0.30	< 0.001
> 0.60, *n* (%)	540 (35.8)	239 (47.0)	301 (30.1)	< 0.001

ROC analysis demonstrated that AI-generated prior-year risk scores modestly discriminated between biopsy-confirmed cancer and non-cancer cases. The AUC was 0.62 (95% CI: 0.59–0.65).

### Associations between prior-year AI risk score and clinical and pathologic characteristics

Among 508 patients with biopsy-confirmed breast cancer, the racial and ethnic distribution included 390 White (76.8%), 54 Black (10.6%), 30 Hispanic (5.9%), and 34 patients (6.7%) identified as other or unknown, as shown in Table 2. The mean (± SD) AI risk score was 1.08 ± 0.52 (median 1.21) at the time of diagnosis, 0.68 ± 0.54 (median 0.57) 1 year prior, and 0.58 ± 0.48 (median 0.41) 2 years prior (*n* = 390).

**Table 2:** Clinical and pathologic characteristics of the study cohort (*N* = 508)

Characteristics	Number (%)
Age (mean ± SD)	66.75 ± 11.06
Race	
White	390 (76.8)
Black	54 (10.6)
Hispanic	30 (5.9)
Other/unknown	34 (6.7)
Breast density	
Fatty	47 (9.3)
Scattered	270 (53.2)
Hetero	175 (34.4)
Extremely dense	16 (3.1)
Cancer centers	
Texas	305 (60.0)
North Carolina	92 (18.1)
Arizona	84 (16.5)
Ohio	27 (5.3)
Pathology	
DCIS	171 (33.7)
Invasive ductal carcinoma	289 (56.9)
Invasive lobular carcinoma	31 (6.1)
Other	17 (3.3)
Receptor types	
HR positive/HER2 negative	222 (43.7)
HR positive/HER2 positive	14 (2.8)
Triple negative	34 (6.7)
HR negative/HER2 positive	3 (0.6)
DCIS HR positive	125 (24.6)
DCIS HR negative	14 (2.8)
Unknown/incomplete profile	96 (18.9)
Tumor grade	
Grade 1	143 (28.1)
Grade 2	214 (42.1)
Grade 3	84 (16.5)
Unknown	67 (13.2)
Case score	Mean ± SD (Median)
Time of diagnosis	78.34 ± 22.55 (86.0)
A year prior	52.94 ± 31.30 (52.0)
Two years prior (*n* = 390)	46.23 ± 28.33 (43.0)
Risk score	Mean ± SD (Median)
Time of diagnosis	1.08 ± 0.52 (1.21)
A year prior	0.68 ± 0.54 (0.57)
Two years prior (*n* = 390)	0.58 ± 0.48 (0.41)

For breast density, 317 (62.4%) were non-dense and 191 (37.6%) were dense. As shown in Table 3, the mean AI risk score was 0.69 ± 0.60 in the dense group, significantly higher than 0.68 ± 0.50 in the non-dense group (*p* = 0.047).

**Table 3:** Associations between clinical and pathologic characteristics and prior-year AI risk scores in univariable linear regression

Characteristics	Risk score (prior year)	β coefficient	95% CI	*p*-value
Age		0.063	(0.052, 0.073)	< 0.0001
Race				
White	0.68 ± 0.53	Ref		
Black	0.71 ± 0.62	0.032	(−0.403, 0.468)	0.884
Hispanic	0.75 ± 0.49	0.297	(−0.272, 0.866)	0.305
Other/unknown	0.69 ± 0.64	−0.106	(−0.642, 0.431)	0.699
Breast density				
Non-dense	0.68 ± 0.50	Ref		
Dense	0.69 ± 0.60	0.277	0.003, 0.551	0.047
Cancer centers				
Texas	0.68 ± 0.55	Ref		
North Carolina	0.78 ± 0.56	−0.189	(−0.557, 0.180)	0.314
Arizona	0.63 ± 0.51	−0.04	(−0.641, 0.560)	0.896
Ohio	0.61 ± 0.47	0.311	(−0.044, 0.667)	0.086
Pathology				
DCIS	0.66 ± 0.56	Ref		
Invasive ductal carcinoma	0.69 ± 0.54	0.261	(−0.026, 0.549)	0.075
Invasive lobular carcinoma	0.90 ± 0.46	0.774	(0.192, 1.355)	0.009
Other	0.48 ± 0.47	−0.15	(−0.908, 0.608)	0.698
Receptor types				
HR positive/HER2 negative	0.72 ± 0.52	Ref		
HR positive/HER2 positive	0.62 ± 0.46	−0.148	(−0.975, 0.678)	0.724
Triple negative	0.61 ± 0.62	−0.373	(−0.925, 0.179)	0.185
HR negative/HER2 positive	1.09 ± 0.60	0.727	(−1.015, 2.470)	0.412
DCIS HR positive	0.67 ± 0.56	−0.314	(−0.649, 0.022)	0.067
DCIS HR negative	0.50 ± 0.54	−0.358	(−1.184, 0.469)	0.396
Unknown/incomplete profile	0.67 ± 0.54	−0.197	(−0.563, 0.169)	0.292
Tumor grade				
Grade 1	0.73 ± 0.51	Ref		
Grade 2	0.70 ± 0.54	−0.205	(−0.527, 0.118)	0.213
Grade 3	0.62 ± 0.59	−0.506	(−0.917, −0.095)	0.016
Unknown	0.63 ± 0.54	−0.209	(−0.651, 0.234)	0.354

Of the 508 patients, 317 (62.4%) had non-dense breasts and 191 (37.6%) had dense breasts. As shown in Table 3, the mean AI risk score was significantly higher in the dense group (0.69 ± 0.60) than in the non-dense group (0.68 ± 0.50) (*p* = 0.047).

**Fig. 1:**
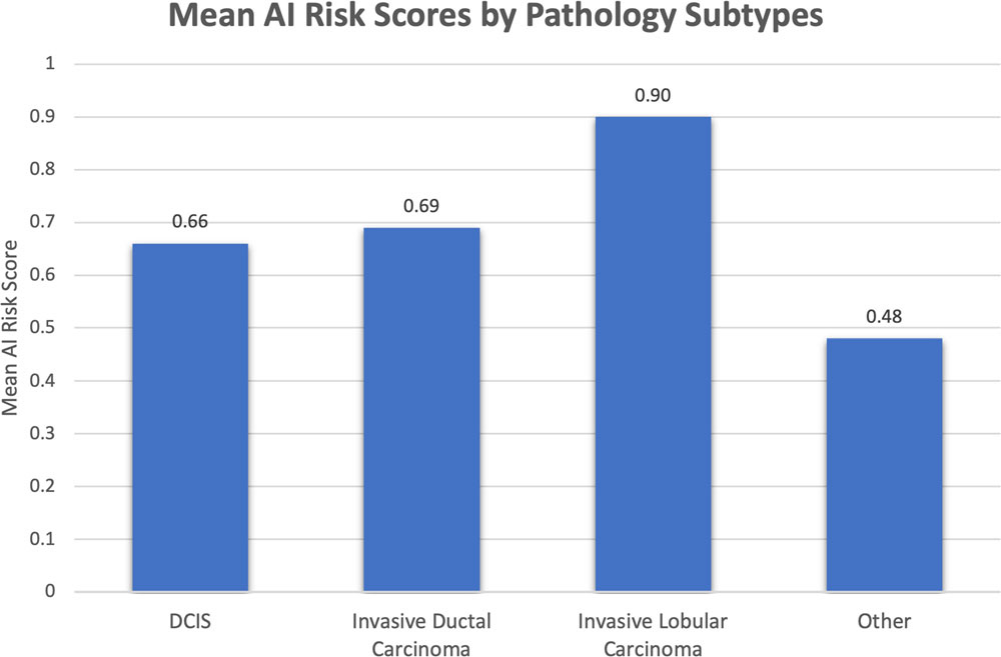
Mean AI risk scores from prior-year screening mammograms by pathology subtypes

For pathology, 171 (33.7%) were diagnosed with DCIS, 289 (56.9%) with IDC, 31 (6.1%) with ILC, and 17 (3.3%) with other invasive carcinoma. As shown in Fig. 1, ILC was associated with the significantly higher risk score (0.90 ± 0.46) (*p* = 0.009), followed by IDC (0.69 ± 0.54), DCIS (0.66 ± 0.56), and other invasive carcinomas (0.48 ± 0.47).

**Fig. 2:**
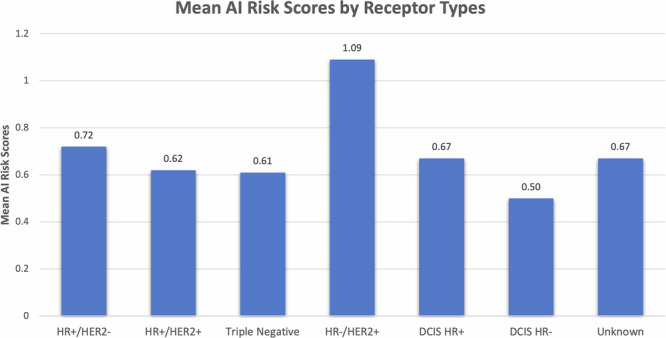
Mean AI risk scores from prior-year screening mammograms by receptor types

The most common receptor subtype was HR-positive/HER2-negative (222, 43.7%), followed by DCIS HR-positive (125, 24.6%). As shown in Fig. 2, HR-negative/HER2-positive cancers had the highest mean AI Risk Score (1.09 ± 0.60), followed by HR-positive/HER2-negative (0.72 ± 0.52). However, these differences did not reach statistical significance, likely due to small sample sizes (Table 3).

**Fig. 3:**
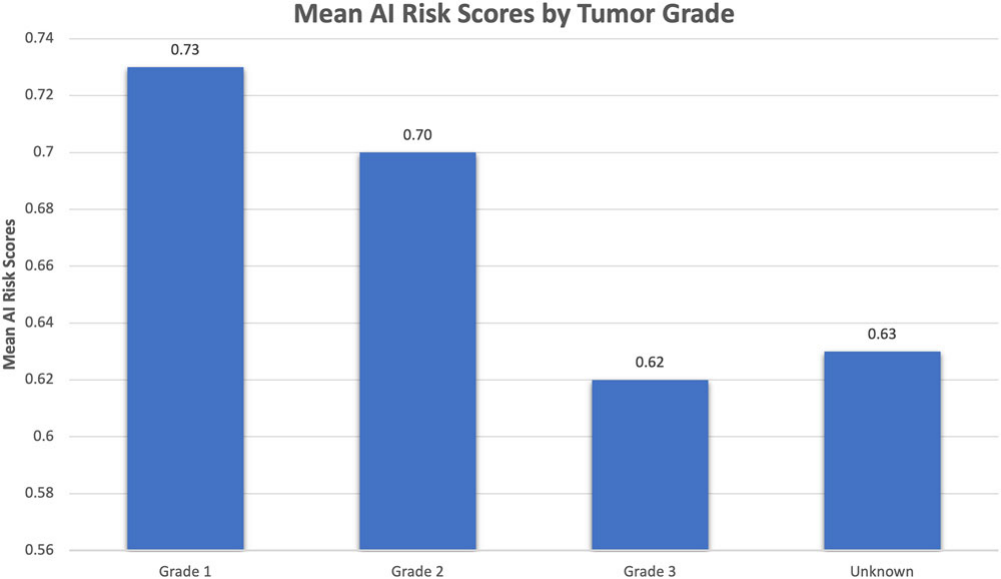
Mean AI risk scores from prior-year screening mammograms by tumor grade

Additionally, the prior-year AI Risk Score was highest for Grade 1 tumors (0.73 ± 0.51), followed by Grade 2 (0.70 ± 0.54) and lowest for Grade 3 tumors (0.62 ± 0.59), as shown in Fig. 3.

Among patients with biopsy-confirmed cancer, univariate linear regression analyses were conducted to assess associations between clinical and pathologic features and prior-year AI risk scores. As expected, age and breast density were significantly associated with a higher prior-year AI risk score. In addition, ILC was associated with significantly higher risk scores compared to DCIS (β = 0.774, 95% CI: 0.192–1.355, *p* = 0.009), and Grade 3 tumors were associated with significantly lower scores compared to Grade 1 tumors (β = −0.506, 95% CI: −0.917 to −0.095, *p* = 0.016), as shown in Table 3.

### Secondary analysis

We further compared clinical and pathologic features between the high-risk (AI risk score > 0.60) and lower-risk (≤ 0.60) groups among the 508 patients with biopsy-confirmed breast cancer. A total of 239 (47.0%) were in the high-risk group, and 269 (53.0%) were in the lower-risk group. As shown in the Supplemental Table, pathologic subtype distribution differed significantly between groups (*p* = 0.007): ILC was more common in the high-risk group (71.0%), while DCIS was more prevalent in the lower-risk group (56.7%). Trends in age, breast density, receptor type, and tumor grade were consistent with findings from the continuous risk score analysis.

### Multivariable linear regression

Two multivariable linear regression models were developed to examine independent predictors of AI Risk Score among the 508 patients with biopsy-confirmed breast cancer, as shown in Table 4. In the first model, adjusted for age, breast density and pathologic subtype, ILC showed a trend toward higher risk scores compared to DCIS, though this did not reach statistical significance (β = 0.476, *p* = 0.075). In the second model, which additionally adjusted for tumor grade, the association between ILC and risk scores was further attenuated and no longer statistically significant (β = 0.407, *p* = 0.136). This suggests that tumor grade may partially mediate the relationship between ILC and elevated AI-predicted risk scores. Notably, Grade 3 tumors showed a marginally significant association with higher AI risk scores compared to Grade 1 tumors (β = 0.476, *p* = 0.068), indicating that tumor grade may contribute to how AI models assess malignancy risk.

**Table 4:** Associations between clinical and pathologic characteristics and prior-year AI risk scores in 2 multivariable linear regression models

Variable	β coefficient	95% CI	*p*-value
Model 1			
Age (per year)	0.063	(0.052, 0.074)	< 0.001
Breast density			
Non-dense	Ref		
Dense	0.128	(−0.127, 0.383)	0.323
Pathologic subtype			
DCIS	Ref		
Invasive ductal carcinoma	0.104	(−0.155, 0.363)	0.43
Invasive lobular carcinoma	0.476	(−0.047, 1.000)	0.075
Other	−0.205	(−0.885, 0.475)	0.554
Model 2			
Age (per year)	0.063	(0.052, 0.074)	< 0.001
Breast density			
Non-dense	Ref		
Dense	0.131	(−0.124, 0.386)	0.314
Pathologic subtype			
DCIS	Ref		
Invasive ductal carcinoma	0.057	(−0.207, 0.322)	0.67
Invasive lobular carcinoma	0.407	(−0.128, 0.943)	0.136
Other	−0.219	(−0.903, 0.465)	0.529
Tumor grade			
Grade 1	Ref		
Grade 2	−0.153	(−0.446, 0.139)	0.302
Grade 3	−0.348	(−0.721, 0.026)	0.068
Unknown	−0.229	(−0.632, 0.175)	0.266

Further descriptive analyses in Table 4 supported this possibility. Patients with ILC were the oldest among all pathology groups (mean age 69.8 ± 9.4 years), while those with DCIS were younger (64.9 ± 10.6 years). Similarly, patients with dense breasts were significantly younger (62.8 ± 11.1 years) than those with non-dense breasts (69.1 ± 10.3 years), reflecting the known inverse relationship between age and breast density. Grade distribution also differed across subtypes. Among the 31 ILC cases, 8 (25.8%) were Grade 1 and 23 (74.2%) were Grade 2, with none classified as Grade 3. This lack of high-grade tumors in ILC may explain the attenuation of the ILC and risk score association after adjusting for tumor grade (Table 5).

**Table 5:** Age and tumor grade distribution by pathologic subtype of breast cancer

Variable	DCIS	Invasive ductal carcinoma	Invasive lobular carcinoma	Other invasive carcinoma
Age (mean ± SD)	64.9 ± 10.6	67.5 ± 11.2	69.8 ± 9.4	66.1 ± 13.4
Tumor grade				
Grade 1	30 (17.5%)	100 (34.6%)	8 (25.8%)	5 (29.4%)
Grade 2	72 (42.1%)	116 (40.1%)	23 (74.2%)	3 (17.6%)
Grade 3	39 (22.8%)	42 (14.5%)	0 (0.0%)	3 (17.6%)
Unknown	30 (17.5%)	31 (10.7%)	0 (0.0%)	6 (35.3%)

## Discussion

Our study found that AI-generated risk scores from prior-year screening mammograms were significantly higher in patients with biopsy-confirmed cancer compared to those with negative biopsies. AI-based risk assessments have emerged as capable of analyzing complex imaging biomarkers that may not be appreciable through traditional models [3, 5, 10, 20]. AI models like Mirai have demonstrated significantly improved discrimination for 5-year risk across international populations, surpassing established tools like Breast Cancer Surveillance Consortium (BCSC) and Tyrer-Cuzick [10, 20–22]. Yet, despite their growing application, the biological relevance of AI-predicted risk scores remained underexplored.

Our study provides insight into how AI-generated risk scores from screening mammograms may reflect underlying tumor biology within a multicenter cohort. In univariable regression, patients with ILC exhibited significantly higher AI risk scores compared to those with DCIS. However, this association was attenuated and lost statistical significance in the multivariable model after adjusting for tumor grade and age. Notably, the majority of ILC cases in our cohort were low grade (Grade 1–2), consistent with the indolent nature of this subtype [23–25], indicating that tumor grade may mediate the relationship between lobular histology and AI-predicted risk. These findings support the possibility that the AI model is sensitive to imaging correlates of tumor differentiation [23, 24].

Additionally, the initially significant association between ILC and higher AI risk scores was attenuated after adjusting for age. This attenuation likely reflects confounding, as ILC was more commonly diagnosed in older women in our cohort. This trend is consistent with prior literature showing that ILC tends to arise in older, postmenopausal women due to its hormone-sensitive biology and distinct tumor evolution pathways [26, 27]. These findings underscore the complexity of interpreting AI risk scores and suggest the model’s ability to capture overlapping imaging signals that reflect both tumor biology and patient demographics.

Another finding from our analysis was the observation of a decreasing trend in AI risk scores with increasing tumor grade. The consistent directionality and near-significant *p*-value suggest a potential inverse relationship between tumor grade and AI-predicted risk. It is likely that lower-grade tumors, due to their slower and more indolent growth, are more readily identified by AI models through subtle texture and compositional imaging features. Prior studies by Gastounioti et al and Zhang et al have shown that AI systems can detect parenchymal and radiomic signatures associated with tumor biology and molecular subtype [28, 29]. This raises the promising possibility of imaging-based prediction of biologic risk that could support tailored breast cancer screening.

In addition, patients with HR-negative/HER2-positive tumors had the highest AI risk scores in our dataset. However, only 3 patients had HR-negative/HER2-positive tumors, and the association did not reach statistical significance. This finding is biologically plausible given the aggressive nature and imaging phenotype of HR-negative/HER2-positive tumors [29–31]. Prior radiomics studies have shown that AI-extracted imaging features correlate with receptor status, tumor grade, and molecular subtype [29, 30, 32, 33]. This trend in our study aligns with the literature, suggesting that with larger samples, statistically robust associations may emerge.

Our results also suggest that AI algorithms may detect age-related imaging features, such as parenchymal involution and compositional shifts, that are not fully captured by traditional density categories. Prior studies have demonstrated that aging is associated with structural changes in the breast parenchyma [28, 34, 35]. Gastounioti et al and Acciavatti et al further emphasized the importance of parenchymal texture, tissue composition, and radiomic features, many of which evolve with age, in imaging-based risk prediction [28, 35]. Collectively, these findings support the notion that AI can recognize and quantify meaningful imaging biomarkers of aging [9, 34].

This study has several limitations. First, the retrospective design restricts our ability to infer causality. In addition, the AUC was calculated using a comparison group of patients with BI-RADS 4–5 findings who underwent biopsy and were found to be negative for cancer. As a result, the imaging contrast between cancer and non-cancer cases could be subtle, which may contribute to the lower AUC and likely underestimates the model’s performance in a general screening population. Second, the relatively small sample sizes for certain subgroups, such as patients with HR-negative/HER2-positive tumors, may have reduced our statistical power to detect significant associations, especially when evaluating the role of tumor biology. Third, for the biopsy-negative group, only age, prior-year AI-generated risk scores, and case scores were collected. This reflected the primary focus of our study, which was to evaluate the association between prior-year AI-derived risk scores and clinical and pathologic characteristics of breast cancer. Fourth, data on interval cancers and prior-year BI-RADS assessments were not available, which may have limited our ability to fully evaluate the longitudinal performance of the AI risk score. Fifth, correction was not performed for multiple comparisons, which may increase the risk of false-positive associations. Sixth, there was missing data for tumor subtype and grade. These patients were categorized as ‘unknown’ and included in the multivariable regression analysis. Finally, although our dataset includes patients from breast imaging centers in four U.S. states and found no significant variation in AI risk scores across racial groups, about three-quarters of the participants were Caucasian, limiting the generalizability of our findings to more diverse populations. Prior studies have underscored the importance of ensuring AI fairness and equitable performance by validating algorithms across demographically diverse populations [36, 37]. Thus, validation in larger, prospective, and demographically diverse cohorts is essential to confirm the robustness of these associations.

In conclusion, AI-generated risk scores from prior-year screening mammograms demonstrated modest ability to discriminate between malignant and non-malignant biopsy outcomes in our multicenter retrospective cohort. Among patients with breast cancer, ILC showed significantly higher prior-year AI risk scores than DCIS in univariate analysis, and Grade 3 tumors showed significantly lower scores than Grade 1. However, after adjusting for tumor grade, the association between ILC and higher risk scores was no longer significant, suggesting that tumor grade may mediate this relationship. Grade 3 tumors showed a marginal association with lower risk scores. These findings suggest that AI risk models may detect subtle imaging patterns indicative of low-grade breast cancers 1 year before clinical detection. Understanding these associations enhances our insight into how AI risk models function and improves their explainability for clinicians. However, future studies are necessary to assess the clinical utility of such models, including whether they improve early detection of aggressive disease or impact clinical outcomes such as treatment intensity, prognosis or survival.

## Supplementary information

Supplementary information

## Abbreviations

AI: Artificial intelligence
AUC: Area under the ROC curve
BI-RADS: Breast Imaging-Reporting and Data System
CI: Confidence interval
DCIS: Ductal carcinoma in situ
ER: Estrogen receptor
FDA: Food and Drug Administration
HER2: Human epidermal growth factor receptor 2
IDC: Invasive ductal carcinoma
ILC: Invasive lobular carcinoma
PR: Progesterone receptor
ROC: Receiver operating characteristic
