# Supplementary material for: From pixels to pathology: how artificial intelligence mammographic risk scores capture tumor biology through imaging
Source: Eur Radiol. 2026 Apr 21;36(8):6730–40. doi: 10.1007/s00330-026-12536-1 (PMC13341831; doi:10.1007/s00330-026-12536-1)
Supplement: Supplementary file 1 — Supplementary information [file 330_2026_12536_MOESM1_ESM.pdf]

From Pixels to Pathology: How Artificial Intelligence Mammographic Risk Scores Capture Tumor Biology Through Imaging

Electronic Supplementary Material

Supplemental Table. Comparison of Clinical and Pathologic Characteristics Between Low (≤0.60) and High (>0.60) Prior-Year AI Risk Score Groups

**Characteristics**	Prior year risk score		p value
	≤0.60	>0.60	
**Age (**Mean ± SD**)**	62.26 ± 10.92	71.80 ± 8.80	<0.001
**Race**			0.863
White	206 (52.8)	184 (47.2)	
Black	30 (55.6)	24 (44.4)	
Hispanic	14 (46.7)	16 (53.3)	
Other/Unknown	19 (55.9)	15 (44.1)	
**Breast Density**			0.284
Fatty	30 (63.8%)	17 (36.2%)	
Scattered	135 (50.0%)	135 (50.0%)	
Hetero	94 (53.7%)	81 (46.3%)	
Extremely Dense	10 (62.5%)	6 (37.5%)	
**Cancer Centers**			0.548
Texas	163 (53.4%)	142 (46.6%)	
North Carolina	43 (46.7%)	49 (53.3%)	
Arizona	48 (57.1%)	36 (42.9%)	
Ohio	15 (55.6%)	12 (44.4%)	
**Pathology**			0.007
DCIS	97 (56.7%)	74 (43.3%)	
Invasive Ductal Carcinoma	150 (51.9%)	139 (48.1%)	
Invasive Lobular Carcinoma	9 (29.0%)	22 (71.0%)	
Other	13 (76.5%)	4 (23.5%)	
**Receptor Types**			0.201
HR positive/HER2 Negative	106 (47.7%)	116 (52.3%)	
HR positive/HER2 Positive	9 (64.3%)	5 (35.7%)	
Triple Negative	21 (61.8%)	13 (38.2%)	
HR negative/HER2 Positive	1 (33.3%)	2 (66.7%)	
DCIS HR Positive	69 (55.2%)	56 (44.8%)	
DCIS HR Negative	11 (78.6%)	3 (21.4%)	

Unknown/Incomplete Profile	52 (54.2%)	44 (45.8%)	
**Tumor Grade**			0.154
Grade 1	67 (46.9%)	76 (53.1%)	
Grade 2	112 (52.3%)	102 (47.7%)	
Grade 3	52 (61.9%)	32 (38.1%)	
Unknown	38 (56.7%)	29 (43.3%)	
**Case Score**			
Time of Diagnosis	31.17 ± 22.94	77.44 ± 18.87	<0.001
Total	269	239	
